# National Continuing Professional Development (CPD) training needs of pharmacists in Ethiopia

**DOI:** 10.1186/s12960-023-00873-x

**Published:** 2023-11-07

**Authors:** Hamere Tamiru, Solomon Assefa Huluka, Bezawit Negash, Kidu Hailu, Zelalem Tilahun Mekonen

**Affiliations:** 1 Cure International Hospital, Addis Ababa, Ethiopia; 2 Department of Pharmacology and Clinical Pharmacy, School of Pharmacy, Addis Ababa University, Addis Ababa, Ethiopia; 3 Department of Pharmaceutics and Social Pharmacy, School of Pharmacy, Addis Ababa University, Addis Ababa, Ethiopia; 4 Ethiopian Pharmaceutical Association, Addis Ababa, Ethiopia

**Keywords:** CPD, Need assessment, Pharmacists, Ethiopia, Training

## Abstract

**Background:**

Continuing Professional Development (CPD) in pharmacy is a lifelong learning approach whereby individual pharmacists are responsible for updating and broadening their knowledge, skills, and attitudes. This is vital to ensure the delivery of high-quality patient care services. However, there is a lack of available data revealing the CPD needs of Ethiopian pharmacists. Thus, the objective of this study was to identify CPD training needs of pharmacists practicing in Ethiopia.

**Methods:**

An institution-based cross-sectional study design with a quantitative approach was employed in this study. This assessment involved 640 pharmacists representing various sectors of the profession. Data were collected through a combination of an online platform and a face-to-face questionnaire administered in person.

**Result:**

A total of 634 participants completed and returned the questionnaires, resulting in an impressive response rate of 99.1%. A significant majority (74.1%) of the participants possessed bachelor’s degree in pharmacy (B. Pharm). *Pharmaceutical Logistics and Pharmacy administration* was preferentially selected as a prior CPD course by 36% of participants, of them while *Pharmacotherapy* (17%), *Leadership/Governance* (13%), Community *Pharmacy* (12%), *Research and Development* (11%) were also the subsequent top choices by participants. Off-site face-to-face lectures (59.2%), Hybrid (face-to-face + e-learning) (54.8%), and on-site on-the-job training (45.5%) were the most convenient means of CPD course delivery. On the other hand, the participants least favored print-based or correspondence programs for CPD course delivery.

**Conclusions:**

CPD holds great importance in the professional lives of pharmacists. It is critical for pharmacists, CPD providers, and those responsible for accrediting CPD programs to recognize the specific CPD requirements, preferred methods of delivery, and obstacles involved. This understanding is vital for establishing priorities and effectively planning CPD activities. In light of this, our study identified the most preferred CPD training courses and convenient delivery methods for pharmacists in Ethiopia. We recommend that CPD providers and accrediting bodies in Ethiopia refer to our findings when approving CPD courses.

## Introduction

Training plays a pivotal role in improving not only skills, attitudes, and knowledge, but also in boosting productivity and the pace of technology adoption[15]. Hence, providing a continuous training is imperative to enhance proficiency. Continuing professional development (CPD) is characterized as an ongoing, systematic, self-directed, structured, outcomes focused cycle of learning and personal growth that occurs throughout individual’s working life. International Pharmaceutical Federation (FIP) further defines CPD as the responsibility of individual pharmacists for systematic maintenance and development of knowledge, skills, and attitudes, to ensure continuing competence as a professional, throughout their careers [19].

CPD has become a crucial aspect of many professions as it helps in reducing the learning curve and accelerating the practical application of knowledge. Rendering an updated and revitalized service in healthcare settings demands a regular upgrading of healthcare professionals’ skills. CPD is instrumental in this regard [20]. The requirements for professionals’ regular engagement in CPD vary across countries and can be mandatory or voluntary [18].

The Ministry of Health of Ethiopia (MoH) is dedicated to enhancing the quality and standards of healthcare services in the country. In its health sector transformation plan, MoH identified strengthening CPD as the key strategic area of Human Resource Development [8]. In line with this, the ministry has issued CPD directive and guideline for healthcare workers, in 2018. Furthermore, in the subsequent year, the ministry integrated the CPD system with health re-licensing process for health professionals, making it mandatory [8, 17].

In Ethiopia, CPD is defined as “*a range of learning activities through which health professionals maintain and develop throughout their career to ensure that they retain their capacity to practice safely, effectively and legally within their scope of practice”* [17].

Pharmacists must be regularly trained in their particular areas of practice in order for them to effectively fulfill their expanding roles in healthcare [22]. With the increasing demand for high-quality healthcare services, patients expect healthcare providers to continuously enhance their professional expertise. Therefore, assessing and understanding the training needs of the workforce becomes essential in instilling confidence and acquiring new skills that enhance preparedness at both individual and team levels within any organization. The ever-changing landscape of technology, persistent workforce shortages, growing disease burden, and resource constraints necessitate healthcare institutions to thoroughly evaluate the performance levels of their staff [2, 12, 17].

Among Ethiopian pharmacists, the most frequently performed task category is the dispensing of medications, which is carried out by 76% of pharmacy professionals. Besides this, pharmacists in Ethiopia are involved in various other areas of practice such as supply chain management, pharmaceutical care, drug information services, regulatory services, and other sectors of the profession [7]. Similar to other countries, the role of Ethiopian pharmacists is still under development and primarily focused on medication dispensing. However, ongoing efforts are being made to amend the practice and academia of the pharmacy sector. This is so as to make the extended role of pharmacy be well established in Ethiopia like other developed nations [10, 14, 16].

The current study aimed to address the paucity of data regarding the CPD needs of Ethiopian pharmacists. As CPD is a new initiative in Ethiopia, it is crucial to generate evidence that identifies the specific CPD needs of pharmacists. This information will aid in the efficient allocation of resources to meet those needs, ensuring that pharmacists receive relevant and tailored education that directly impacts their professional practice [12, 17]. The Ethiopian Pharmaceutical Association, in collaboration with partners, has taken the initiative to identify the CPD needs of its members. This effort is essential for the advancement of the pharmaceutical sector in the country and ensuring that pharmacists are equipped with the necessary skills and knowledge to provide quality healthcare services. By conducting this study, the aim is to gather evidence that will guide the development and implementation of CPD programs for pharmacists in Ethiopia.

## Methods

### Study area

According to the national human resource update, Ethiopia, Africa’s second most populated country, has employed 342 899 heath workers in public health facilities. Out of this total, pharmacists are expected to make up 5%, which is approximately 17 091 pharmacists [5]. Pharmacists from various sectors working in seven regions (Amhara, Oromia, SNNPR, Sidama, Benishangul-Gumuz, Harari, and Somali) and two city administrations (Addis Ababa and Dire-Dawa) were considered in this national CPD need assessment. For the reason stated, regions with security problem (Tigray and Afar) and one region with a relatively very small number of pharmacists (Gambella) were excluded.

### Study design and period

From July 20, 2021 to August 20, 2021, an institution-based cross-sectional study design was employed in our study. Quantitative data were collected using both face to face and online surveys.

### Sampling procedure and assumptions

The sample size for the study population was determined using a single proportion formula was used to calculate the sample size of the study population, with a confidence level of 95%, a margin of error of 5%, and a presumed proportion of 0.05 (*P* = 0.05). In addition, a design effect of 1.5 was considered to minimize the effect of the design with multi-stage stratified sampling. Initially, the calculated sample size was 634, assuming a non-response rate of 10%. However, to adjust for fractional numbers in the stratified sampling, the final sample size was increased to 640. The samples were then proportionally allocated to the respective strata of the health administration structure and practice area.

It is important to note that all participants responded to the majority of the questionnaires. For variables with more than two missing values, listwise deletion was used. For other variables, mean substitution was used whenever applicable. Additionally, each item had a predefined minimum and a maximum score of within the given range, ensuring that there were no errors in the data.

### Data collection process

A total of 16 data collectors, each holding at least a Bachelor of Pharmacy degree and 4 supervisors with a minimum of Master’s degree, were involved in the data collection process. To ensure that the data collectors were familiar with the tools, a one-day virtual training was provided to them. Subsequently, data collectors conducted a pre-test on the Google Survey platform. The data were collected in a dual modality. For the nearby participants, the data were collected using face-to-face administered questionnaire. For other participants at a distance, a Google Survey link was shared, allowing them to conveniently respond on their chosen platforms. Study coordinators were assigned in the respective regions to facilitate both the online and face-to-face data collection. Supervisors were responsible for ensuring the completeness and adequacy of the collected data. They checked the gathered data on a daily basis and addressed any identified issues or problems to ensure the accuracy and quality of the data. Corrections were made as necessary under the supervision of the supervisors.

### Data quality assurance

In order to ensure the accuracy and reliability of the gathered data, a series of measures were taken. These included subjecting the data collection tool to scrutiny by multiple experts, conducting a pre-test to identify and correct any issues beforehand, providing training to all data collectors to ensure consistency in data collection, and having coordinators verifying the completeness of the data for their respective clusters.

### Data management and analysis

Collected data were primarily checked for its completeness and internal consistency. Afterwards, the data were exported from the Google Survey Platform to excel sheet and imported to SPSS Version 26 for data management and analysis.

### Ethical consideration

Ethical clearance letter was secured from the Ethical Review Committee of Yekatit 12 Hospital Medical College (Protocol no. 91/21) prior to the commencement of the study. Informed written consent was obtained from all participants after a detail explanation of study’s purpose and procedure. Any kind of individual identifiers were not included in the data collection tool to protect the privacy of the participants. The collected data were analyzed and reported in aggregate form, further safeguarding the participants' privacy and anonymity.

## Results

### Participants’ characteristics

A total of 640 individuals were approached, and out of these, 634 (99.06%) of them completed the questionnaire. Majority of the study participants were male, accounting for 82.5% of the total respondents. Additionally, 74.1% of the participants held a Bachelor's degree (Table 1).

**Table 1:** Socio-demographic characteristics of study participants, Ethiopia, August 2021

Variables (*N* = 634)	Frequency	Percentage (%)
Gender		
Male	523	82.5
Female	111	17.5
Highest level of education		
B. Pharm	470	74.1
MSc/M. Pharm	134	21.1
MBA	8	1.3
MA	12	1.9
MPH	7	1.1
PhD	3	0.5
Work experience		
< 5 years	177	27.9
6–10 years	196	30.9
> 10 years	259	40.9
Region		
Addis Ababa	145	22.9
Oromia	162	25.6
Amhara	192	30.3
SNNPR	43	6.8
Sidama	48	7.6
Somali	16	2.5
Diredewa	5	0.8
Harrari	9	1.4
Benishanul Gumuz	14	2.2

*SNNPR* Southern Nation, Nationalities and Peoples’ Regional state

### Pharmacists topic preference across CPD domains of competency

**Fig. 1:**
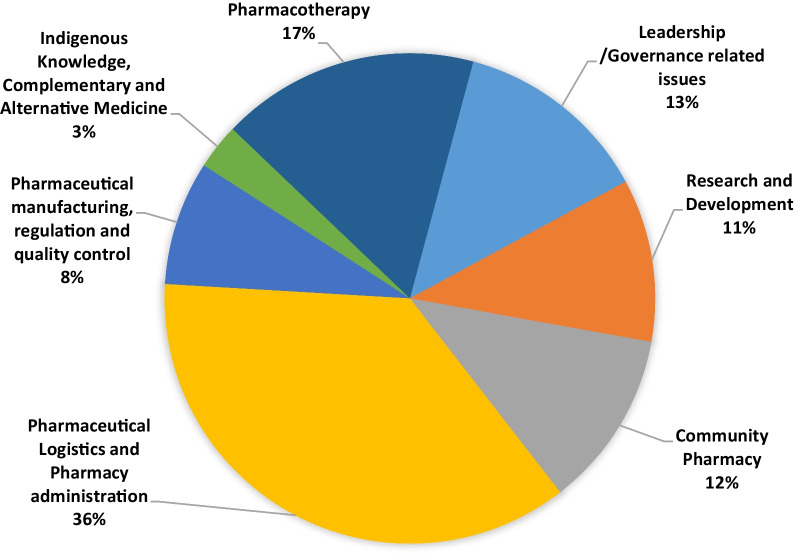
Pharmacists’ preference to topics of CPD domains of competency, August 2021, Ethiopia

The CPD topics were organized into domains of competency. According to participant preferences, the top three selected topics were *Pharmaceutical Logistics and Pharmacy Administration* (36%), *Pharmacotherapy* (17.1%), and *Leadership/Governance* related issues (13%) (Fig. 1).

### Pharmacists’ preference to CPD topics across different organization

Overall, the selection of CPD topics across different organizations was deemed to be closely aligned to their respective working sector. Of the total 92 participants who are working in the pharmaceutical industry/import/wholesale sectors, nearly half of them (48.9%) opted CPD topics in the domain of *Pharmaceutical Logistics and Pharmacy administration*. Likewise, out of the total 75 respondents working either in private or public community pharmacies, 30 (40%) of them preferred *community pharmacy* related topics. Similarly, when considering the 225 pharmacists practicing in public and private hospital pharmacies, 40% (90 participants) showed a preference for topics in the domain of *Pharmaceutical Logistics and Pharmacy administration* (Table 2).

**Table 2:** Pharmacists’ preference to CPD topics across different organizations, August 2021, Ethiopia

Organizations	Leadership/governance-related issues *N* (%)	Research and development *N* (%)	Community pharmacy *N* (%)	Pharmaceutical logistics and pharmacy administration *N* (%)	Pharmaceutical manufacturing, regulation, and quality control *N* (%)	Indigenous knowledge, complementary and alternative medicine *N* (%)	Pharmacotherapy *N* (%)	Total *N*
FMOH/RHB/ZHD/WoHO	22 (20.6)	6 (5.6)	9 (8.4)	51 (47.7)	5 (4.7)	5 (4.7)	9 (8.4)	107
Pharmaceutical industry/ import/wholesale	9 (9.8)	9 (9.8)	6 (6.52)	45 (48.9)	13 (14.1)	2 (2.1)	8 (8.7)	92
Public/private hospital pharmacy and health facility	25 (11.1)	18 (8)	21 (9.3)	90 (40.0)	17 (7.5)	1 (0.5)	53 (23.5)	225
Public/private community pharmacy	4 (5.3)	4 (5.3)	30 (40.0)	12 (16.0)	4 (5.3)	3 (4.0)	18 (24.0)	75
Local/international non-governmental organization	7 (26.9)	4 (15.4)	3 (11.5)	6 (23.1)	2 (7.7)	2 (7.7)	2 (7.7)	26
Public/private academic institutions	5 (8.9)	21 (37.5)	2 (3.6)	8 (14.3)	2 (3.6)	5 (8.9)	13 (23.2)	56
Regulatory institution and others	10 (19.6)	5 (9.8)	3 (5.88)	18 (35.3)	9 (17.64)	1 (1.9)	5 (9.8)	51
Column total	82 (13.0)	67 (10.6)	74 (11.7)	230 (36.4)	52 (8.2)	19 (3.0)	108 (17.1)	632 (100%)*

*Slight data deviation can be observed due to missing data

*FMOH* Federal Ministry of Health, *ZHD* Zonal Health Department, W*oHO* Woreda Health Office

### Pharmacists’ perception on the level of CPD training need

**Fig. 2:**
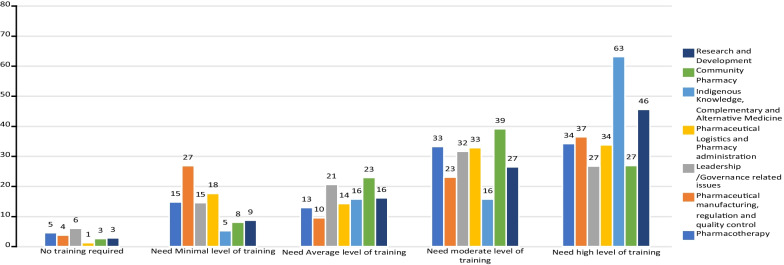
Pharmacists’ perception on the level of CPD training need in the respective competency areas in Ethiopia, August 2021

Participants were requested to rank their self-perceived CPD training needs using a 5-point scale with ordinal categories. High level of training need for CPD was claimed for “*Indigenous knowledge, complementary and alternative medicine”* and “*Research and development”* as indicated by 63 and 46 of the participants, respectively. Although there is a high total number of participants who selected the topic of pharmaceutical logistics and pharmacy administration, their perception of training need is quite evenly distributed between high (34) and moderate (33) levels of training (Fig. 2).

### Pharmacists’ preference on CPD delivery methods

**Fig. 3:**
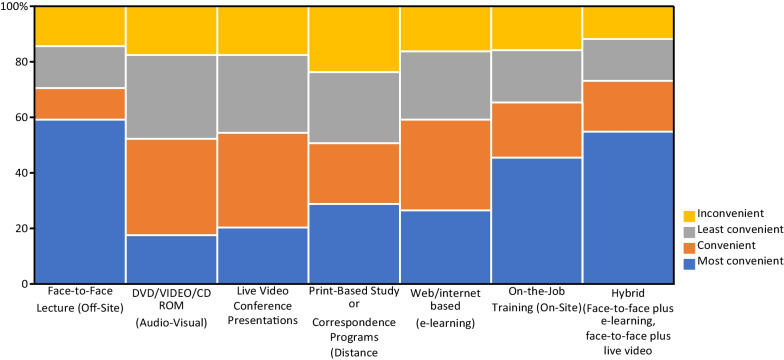
Pharmacists’ preference on CPD delivery methods in Ethiopia, August 2021

Participants were asked to rank their preferences for delivery method for the CPD courses. The top rated most convenient CPD delivery modes, in rank order, were off-site face-to-face lectures (59.2%), hybrid (face-to-face plus e-learning) (54.8%), and on-site on-job training (45.5%). Print-based study or correspondence programs were the least preferred method of delivery of CPD courses (Fig. 3).

**Fig. 4:**
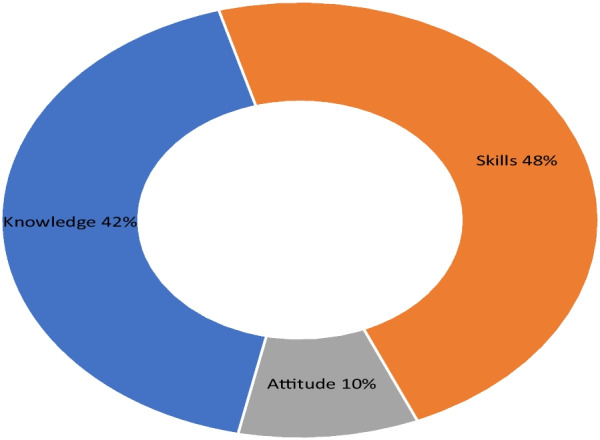
Pharmacists’ preference on CPD competency domains in Ethiopia, August 2021

Regarding the preference for learning competency domains, there was a notable difference. Skill-based training was the most preferred domain, chosen by 48% of respondents, followed by knowledge-based training at 42%. The least preferred learning domain was attitude-based training, mentioned by only 10% of respondents (Fig. 4). The major reported barriers to lifelong learning for pharmacists were the absence of IT infrastructure/computer literacy (60.6%), followed by absence of standards of practice and a performance monitoring system (50.6%).

## Discussion

According to a census conducted in Ethiopia in 2010, majority of pharmacists are practicing in hospital, sales and marketing and community pharmacies [11]. In these settings “*Pharmaceutical logistics and pharmacy administration*” has been identified as the core activity [24]. This is in congruent with the present study in which the top rated CPD topic preference for pharmacists was “*Pharmaceutical Logistics and Pharmacy Administration*”. Since many pharmacists are involved in pharmaceutical supply chain management and dispensing activities, developing CPD training materials in the area will improve medicines availability and enhance the quality of pharmacy service [6].

This study demonstrated a significant relation between CPD topic preferences and the type of organization where pharmacists work. For instance, large percentage of pharmacists working in the community pharmacy expressing a preference for pursuing CPD courses in the community pharmacy related areas. Although this study involves pharmacists from different practice area, it is worth noting that soft-skills within the leadership/governance domain were selected as one of the top three preferred area of CPD topics. Likewise, a qualitative study done in Toronto with 20 community pharmacists designed to elicit learning needs for scope of practice. The study found that pharmacists have access to hard sciences, but they often miss opportunities for soft skills development. [1].

In this study, the majority of participants expressed a preference for skill-based learning, followed by knowledge-based learning. However, a study done in India using a mixed study design showed a low response rate for knowledge items (ranging from 3 to 9.3%) and low practice level (ranging from 2.4 to 33.8%) in terms of patient-oriented services, whereas attitude to provide patient care services was high, ranging from 80 to 100%) [21].

Considering that knowledge and skill are interconnected, it is recommended to incorporate skill development, as well as the disposition and competence of Ethiopian pharmacists, into future training programs. By doing so, pharmacists can enhance their potential impact on healthcare outcomes.

One of the major barriers claimed by participants for CPD training was absence of IT infrastructure/computer literacy. A review of barriers to CPD in radiography in Africa also showed attitudinal, physical and structural barriers [3]. As e-learning requires IT infrastructure/literacy, this will be a critical challenge for delivering virtual trainings. In a study conducted in north western Ethiopia, lack of relevant learning opportunities, inaccessibility, cost and family constraints were mentioned as a barrier for CPD training [12]. Those mentioned barriers might influence their preference to mode of CPD delivery. The second barrier for lifelong learning in this study was absences of standard practice and performance monitoring. Similarly, in a cross-sectional study done in selected Africa countries, majority of the countries did not have a validated competency framework for early career pharmacy practice [23].

In this study, pharmacists claimed face-to-face lecture as the preferred mode of delivery, whereas print-based studies (correspondence programs) were their least favored. Similarly, pharmacists favored interactive talks as a mode of delivery in a study done in northern Ethiopia. Other research has also shown that interactive tactics, such as academic details and audit/feedback, tend to be more effective, whereas print media, such as self-study posters, are generally ineffectual [9]. In contrast, a study conducted in United Arab Emirates on the continuing education needs of pharmacists revealed that majority of them expressed high preference for computer and internet-based formats, followed by live-in person and printed material-based programs [13]. Additionally, it has been found that using multiple delivery methods for CPD tends to yield more positive results than relying on a single method [4]. Therefore, it is recommended that CPD trainings incorporate a variety of modalities in order to cater to different learning preferences and domains of pharmacists.

## Conclusion

The study revealed that majority of pharmacists prefer to learn pharmaceutical logistics and pharmacy administration followed by pharmacotherapy and leadership and governance-related issues. The finding further showed the existence of significance relationship between CPD topic preference and the type of organization where pharmacists work. It is important to note that need assessment should not be taken as an end goal on its own. Rather, the information gathered must be put to good use in order to meet those needs.

Understanding CPD need is important to CPD providers and accreditors to identify the priority CPD need of pharmacists, the challenges to lifelong learning, and preferred delivery method and programs. Based on the findings, CPD accreditors and Providers in Ethiopia can prioritize the development of CPD courses.

## Limitation of the study

The study did not address the CPD training needs of pharmacy professionals with qualifications below a bachelor's degree. Furthermore, the preferred assessment methods of the courses were not extensively studied in the current research. These aspects could be considered as potential areas for future studies and research to further explore and understand the CPD training needs and assessment preferences of pharmacy professionals with all levels of qualifications.

## Data Availability

Not applicable.

## Abbreviations

CPD: Continuing Professional Development
FIP: International Pharmaceutical Federation
MOH: Ministry of Health of Ethiopia
SNNPR: South Nations, Nationalities and People’s Region
SPSS: Statistical Package for Social Science
HWIP: Health Work Force Improvement Program

## References

[1] Austin Z, Gregory P (2019). Learning needs of pharmacists for an evolving scope of practice. Pharmacy.

[2] Batista JPB, Torre C, Sousa Lobo JM, Sepodes B (2022). A review of the continuous professional development system for pharmacists. Hum Resour Health.

[3] Bwanga O (2020). Barriers to continuing professional development (CPD) in radiography: a review of literature from Africa. Health Professions Education.

[4] Davis D, Galbmith R (2009). Continuing medical education effect on practice performance. Chest.

[5] DHET. Annual performance. October, 162. 2021. www.treasury.gov.za/publications/.../NT.APP.2014-18.pdf

[6] FIP. (2002). International Pharmaceutical Federation Statement of professional standards on continuing professional development. 1993, 1–4. http://www.fip.org/www/uploads/database_file.php?id=221&table_id=. Accessed Apr 1997.

[7] Federal Ministry of Health. Ethiopia task analysis study report: for Medical Doctors, Health Officers, Nurses, Medical Laboratory Professionals, and Pharmacy Professionals. 2015

[8] FMOH (2021). Health sector transformation plan II 2020/2021-2024/2025. Ethiop Ministry Health.

[9] Forsetlund L, Bjørndal A, Rashidian A, Jamtvedt G, O’Brien MA, Wolf F, Davis D, Odgaard-Jensen J, Oxman AD (2009). Continuing education meetings and workshops: Effects on professional practice and health care outcomes. Cochrane Database oSyst Rev.

[10] García-Cárdenas V, Sabater-Hernández D, Kenny P, Martínez-Martínez F, Faus MJ, Benrimoj SI (2013). Effect of a pharmacist intervention on asthma control. A cluster randomized trial. Respir Med.

[11] Gebretekle GB, Fenta TG (2010). Assessment of the pharmacist workforce in Ethiopia. Ethiop J Health Dev.

[12] Gelayee DA, Mekonnen GB, Birarra MK (2018). Involvement of community pharmacists in continuing professional development (CPD): A baseline survey in Gondar, Northwest Ethiopia. Global Health.

[13] Hasan S (2009). Continuing education needs assessment of pharmacists in the United Arab Emirates. Pharm World Sci.

[14] Khdour MR, Kidney JC, Smyth BM, McElnay JC (2009). Clinical pharmacy-led disease and medicine management programme for patients with COPD. Br J Clin Pharmacol.

[15] Mahmud KT, Saira Wahid I, Arif I (2019). Impact of training needs assessment on the performance of employees: evidence from Bangladesh. Cogent Soc Sci.

[16] Marra C, Johnston K, Santschi V, Tsuyuki RT (2017). Cost-effectiveness of pharmacist care for managing hypertension in Canada. Can Pharm J.

[17] Ministry F. Federal ministry of health Ethiopia directive on continuing professional development for health professionals. 2018

[18] Mlambo M, Silén C, McGrath C (2021). Lifelong learning and nurses’ continuing professional development, a metasynthesis of the literature. BMC Nurs.

[19] Naoko Arakawa SY. Japanese Pharmacists Association Lifelong Learning Support System, on-line portfolio system. Int Pharm Federation (FIP), 2014:1–46. http://www.fip.org/files/fip/PharmacyEducation/CPD_CE_report/FIP_2014_Global_Report_CPD_CE_online_version.pdf

[20] Pool IA, Poell RF, Berings MGMC, ten Cate O (2016). Motives and activities for continuing professional development: an exploration of their relationships by integrating literature and interview data. Nurse Educ Today.

[21] Pawar SB, Pawar AP (2018). Training need assessment of dispensing pharmacists in Maharashtra. Int J Pharm Sci Res.

[22] Tofade T, Duggan C, Rouse M, Anderson C (2015). The responsibility of advancing continuing professional development and continuing education globally. Am J Pharm Educ.

[23] Udoh A, Bruno A, Bates I (2018). A survey of pharmacists’ perception of foundation level competencies in African countries. Hum Resour Health.

[24] WHO. The role of pharmacist in the health care system 1994; p. 59.

